# Aortite thoraco-abdominale révélant une maladie de Horton

**DOI:** 10.11604/pamj.2014.18.139.4776

**Published:** 2014-06-17

**Authors:** Wafa Chebbi, Saida Jerbi

**Affiliations:** 1 Service de Médecine Interne, CHU Taher Sfar Mahdia, 5100 Mahdia, Tunisie; 2 Service de Radiologie, CHU Taher Sfar Mahdia, 5100 Mahdia, Tunisie

**Keywords:** Maladie de Horton, aortite inflammatoire, Horton disease, inflammatory aortitis

## Image en medicine

La maladie de Horton est une vascularite segmentaire et focale qui touche les artères de gros et moyen calibre, intéressant préférentiellement les branches de l'artère carotide externe. L'atteinte aortique est observée dans 5 à 15% des cas, mais elle est probablement sous-estimée en raison d'une symptomatologie souvent pauvre ou peu spécifique. Elle est rarement inaugurale et survient le plus souvent lors de la décroissance de la corticothérapie. Nous rapportons l'observation d'une patiente âgée de 75 ans, hospitalisée pour exploration d'une fièvre prolongée associée à un syndrome inflammatoire biologique. A l'anamnèse, la patiente rapportait des céphalées fronto-pariétales depuis un mois. Il n'y avait pas de notion d'altération de l’état général, ni de sueurs nocturnes, ni de signe de pseudo polyarthrite rhizomélique. L'examen clinique était normal. Les pouls temporaux étaient pulsatiles. Le bilan biologique montrait une anémie à 8,6 g/l, une vitesse de sédimentation à 132 mm à la première heure, une protéine C réactive à 68 mg/l, une fibrinémie à 8,8 g/l et une hyper-alpha2-globulinémie. Les anticorps antinucléaires et les ANCA étaient négatifs. La recherche d'un foyer infectieux pulmonaire, ORL, stomatologique et urinaire était négative. L’échographie transœsophagienne ne montrait pas des signes en faveur d'une dissection aortique ou d'une endocardite. En l'absence de point d'appel, une tomodensitométrie thoraco-abdominale a été réalisée, mettant en évidence un épaississement pariétal régulier et circonférentiel intéressant l'aorte thoracique, les troncs supra-aortiques, l'aorte abdominale et les artères iliaques primitives à leur origine. La biopsie d'artère temporale montrait un aspect d'artérite temporale à cellules géantes. Un traitement par corticoïdes à raison de 1 mg/kg/j était instauré, entrainant une disparition rapide de la fièvre et des céphalées et une régression du syndrome inflammatoire.

**Figure 1:**
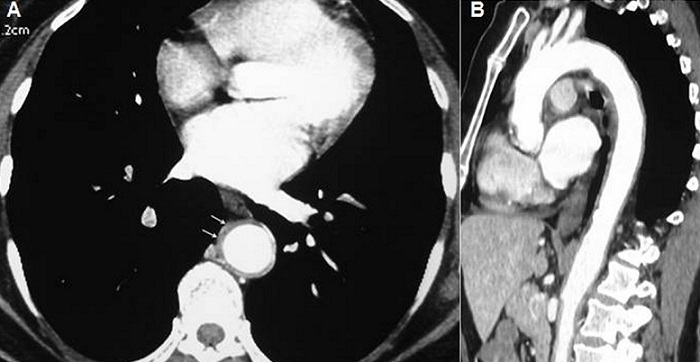
TDM thoraco-abdominale dans le plan axial (A) et en reconstruction coronale; (B) Épaississement pariétal circonférentiel et régulier de l'aorte thoracique et abdominale (flèches)

